# Low-Avidity Autoantibodies against Bactericidal/Permeability-Increasing Protein Occur in Gram-Negative and Gram-Positive Bacteremia

**DOI:** 10.1128/IAI.00444-20

**Published:** 2020-09-18

**Authors:** J. Theprungsirikul, J. T. Thaden, R. M. Wierzbicki, A. S. Burns, S. Skopelja-Gardner, V. G. Fowler, K. L. Winthrop, I. W. Martin, W. F. C. Rigby

**Affiliations:** a Department of Microbiology and Immunology, Geisel School of Medicine at Dartmouth, Lebanon, New Hampshire, USA; b Division of Infectious Diseases, Department of Medicine, Duke University School of Medicine, Durham, North Carolina, USA; c Center for Infectious Disease Studies, OHSU-PSU School of Public Health, Portland, Oregon, USA; d Department of Pathology and Laboratory Medicine, Geisel School of Medicine at Dartmouth, Lebanon, New Hampshire, USA; e Division of Rheumatology, Department of Medicine, Geisel School of Medicine at Dartmouth, Lebanon, New Hampshire, USA; Georgia Institute of Technology School of Biological Sciences

**Keywords:** bacteremia, autoreactivity, bactericidal/permeability-increasing protein, BPI, bronchiectasis, *Pseudomonas*, *Pseudomonas aeruginosa*, autoimmunity

## Abstract

Antibody autoreactivity against bactericidal/permeability-increasing protein (BPI) is strongly associated with Pseudomonas aeruginosa infection in cystic fibrosis (CF), non-CF bronchiectasis (BE), and chronic obstructive pulmonary disease (COPD). We examined the pathogen-specific nature of this autoreactivity by examining antibodies to BPI in bacteremia patients. Antibodies to BPI and bacterial antigens were measured in sera by ELISA from five patient cohorts (*n* = 214). Antibody avidity was investigated. Bacteremic patient sera (*n* = 32) exhibited IgG antibody autoreactivity against BPI in 64.7% and 46.7% of patients with positive blood cultures for P. aeruginosa and Escherichia coli, respectively. Autoantibody titers correlated with IgG responses to bacterial extracts and lipopolysaccharide (LPS). A prospective cohort of bacteremic patient sera exhibited anti-BPI IgG responses in 23/154 (14.9%) patients with autoreactivity present at the time of positive blood cultures in patients with Gram-negative and Gram-positive bacteria, including 8/60 (13.3%) patients with Staphylococcus aureus. Chronic tissue infection with S. aureus was associated with BPI antibody autoreactivity in 2/15 patients (13.3%). Previously, we demonstrated that BPI autoreactivity in CF patient sera exhibits high avidity. Here, a similar pattern was seen in BE patient sera. In contrast, sera from patients with bacteremia exhibited low avidity. These data indicate that low-avidity IgG responses to BPI can arise acutely in response to bacteremia and that this association is not limited to P. aeruginosa. This is to be contrasted with chronic respiratory infection with P. aeruginosa, suggesting that either the chronicity or the site of infection selects for the generation of high-avidity responses, with biologic consequences for airway immunity.

## Abstract (precis)

Antibody autoreactivity against bactericidal/permeability-increasing protein (BPI) is strongly associated with Pseudomonas aeruginosa infection in cystic fibrosis (CF), non-CF bronchiectasis (BE), and chronic obstructive pulmonary disease (COPD). We examined the pathogen-specific nature of this autoreactivity by examining antibodies to BPI in bacteremia patients. Antibodies to BPI and bacterial antigens were measured in sera by ELISA from five patient cohorts (*n* = 214).

## INTRODUCTION

Recurrent infections and subsequent airway damage are major causes of morbidity and mortality in bronchiectasis (BE), chronic obstructive pulmonary disease (COPD), and cystic fibrosis (CF). The Gram-negative bacterium Pseudomonas aeruginosa is a major pathogen in each of these diseases (1–4). Neutrophils are critical early responders to P. aeruginosa lung infection. Neutrophil depletion or altered functions results in a marked increase in P. aeruginosa susceptibility (reviewed in reference 5). Recurrent P. aeruginosa infections lead progressively to worsening of lung function. Moreover, autoantibodies to bactericidal/permeability-increasing protein (BPI), a potent antimicrobial agent made by neutrophils (6, 7), strongly associate with P. aeruginosa infection and inversely correlate with lung function in BE, CF, and COPD (3, 4, 8–10). The strength of this relationship suggests a model by which P. aeruginosa exhibits a seemingly selective ability to cause autoantibody development to BPI, thereby compromising innate immune responses and leading to its chronic airway infection.

BPI is a cationic protein of ∼55 kDa and is stored in primary azurophilic granules of neutrophils. Upon neutrophil activation, BPI is released to mediate bactericidal effects due to its high-affinity binding to the lipopolysaccharide (LPS) selectively present in Gram-negative bacteria (GNB) (7, 11–17). BPI additionally opsonizes targeted bacteria for enhanced phagocytosis and neutralizes the inflammatory effects of LPS by reducing LPS-mediated neutrophil stimulation and tumor necrosis factor alpha (TNF-α) production (15, 18). BPI consists of an N-terminal domain, a C-terminal domain, and a proline-rich linker that connects the two domains together (19). The LPS binding site is located in the N-terminal domain of BPI, which consists of hydrophilic and basic residues mapped to amino acids 1 to 220 (19, 20). BPI and other bactericidal proteins released from activated neutrophils may independently contribute to host tissue damage and dysfunction, together with the release of proteases, reactive oxygen species (ROS), and reactive nitrogen species (RNS) (21, 22). These cascades leading to tissue injury may possibly prompt the host to generate autoantibodies against BPI.

One possible explanation for the association between P. aeruginosa and BPI autoantibody generation is molecular mimicry, where P. aeruginosa antigens induce antibodies directed not only against P. aeruginosa but also against the self-protein BPI. We test our speculation by investigating whether acute, rather than chronic P. aeruginosa, types of bacterial infections (Gram-negative versus Gram-positive) might lead to anti-BPI antibody induction and whether anti-BPI antibody responses in various infections are qualitatively different and mediated by pathophysiologic pathways.

In this study, we tested anti-BPI IgG induction in patients with Gram-negative bacteremia (P. aeruginosa and Escherichia coli). Surprisingly, comparable frequencies of IgG anti-BPI autoantibodies were seen in patients with P. aeruginosa and Escherichia coli bacteremia at a frequency that paralleled humoral responses to bacterial proteins and LPS. This lack of a specific requirement for P. aeruginosa for anti-BPI autoantibody induction was confirmed in a consecutive cohort of bacteremic patients, in which anti-BPI IgG was also found in Staphylococcus aureus infection. Remarkably, these latter studies demonstrated rapid IgG autoantibody induction against BPI contemporaneous with, or shortly after, the onset of bacteremia. Subsequent studies demonstrated that these IgG autoantibody responses are of low avidity, in contrast to those seen in BE patients with P. aeruginosa. The implications of these findings include the generation of two qualitatively different autoimmune responses against BPI as a function of different disease states and/or chronicity. These findings are considered in the context of the development of autoantibodies to BPI in disease states other than chronic airway infection.

## RESULTS

### BPI autoreactivity in Gram-negative bacteremia.

**FIG 1:**
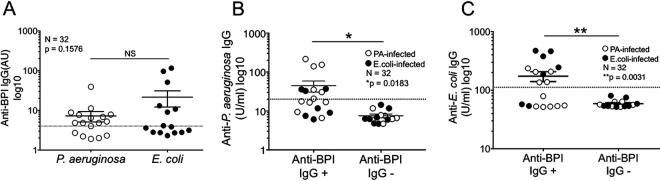
Anti-BPI IgG antibodies in patients with P. aeruginosa and E. coli bacteremia. (A) Anti-BPI IgG titers detected by ELISA in the Duke bacteremia cohort: P. aeruginosa bacteremia (*n* = 17, 64.7% positive, 7.2 ± 2.095 AU [mean ± standard error of the mean {SEM}]) and E. coli bacteremia (*n* = 15, 46.7% positive, 21.49 ± 9.391 AU [mean ± SEM]); anti-BPI IgG positivity is indicated by >4 AU. (B and C) Relationship between anti-BPI IgG positivity and antibody reactivity to P. aeruginosa (PA14 lysate) (B) and E. coli (GN02546 lysate) (C) in the Duke bacteremia cohort (*n* = 32); reactivity to P. aeruginosa and E. coli was determined by ELISA (positive cutoffs of >22 U/ml and >112 U/ml, respectively). Positive cutoffs were determined as the mean values for healthy controls plus 2 standard deviations (SD) (*n* = 53) and are represented by dashed lines. Filled symbols represent E. coli bacteremic patient sera, and unfilled symbols represent P. aeruginosa bacteremic patient sera, by blood culture. Statistical significance was determined by Student's *t* test (*, *P* < 0.05; **, *P* < 0.01). NS, not significant.

We previously reported an association between autoreactivity to BPI and chronic P. aeruginosa infection in the serum of CF and non-CF BE patients (9, 10). We evaluated if this relationship extended to patient sera with P. aeruginosa bacteremia infection (*n* = 17; see Duke bacteremia cohort characteristics in Table 1). Sera from patients with E. coli bacteremia (*n* = 15) were used as controls for the specificity of these responses for P. aeruginosa. Healthy control sera exhibited little reactivity against PA14 (12.9 ± 4.51 U/ml) and E. coli (70.3 ± 20.9 U/ml) extracts, as well as BPI (2.54 ± 0.75 arbitrary units [AU]) (data not shown). Anti-BPI autoantibodies were present in 64.7% (11/17) and 46.7% (7/15) of P. aeruginosa- and E. coli-infected patients, respectively, with a biphasic distribution (Fig. 1A). The mean level of IgG anti-BPI antibodies in positive patients was greater in E. coli patient sera (21.49 ± 9.391 AU) than in P. aeruginosa patient sera (7.2 ± 2.095 AU). We thus conclude that bacteremia caused by both GNB can induce IgG responses to BPI, arguing against specific molecular mimicry triggered by P. aeruginosa in this setting.

**TABLE 1:** Patient cohort characteristics^*a*^

Cohort (*n*)	Disease (acuity)	Infecting organism(s)	No. of patients	Time of serum collection (days) (mean ± SD)
Duke bacteremia (32)	Bacteremia (acute)	Pseudomonas aeruginosa	17	NA
		Escherichia coli	15	NA
DHMC bacteremia (154)	Bacteremia (acute)	Staphylococcus aureus	60	9.3 ± 12.3
		Escherichia coli	29	6.0 ± 7.2
		Pseudomonas aeruginosa	12	10.9 ± 19.6
		Klebsiella pneumoniae	10	4.2 ± 2.4
		Enterococcus faecalis	3	3.3 ± 2.5
		Others^*b*^	40	5.4 ± 3.8
DHMC bronchiectasis (3)	Bronchiectasis (chronic)	Pseudomonas aeruginosa	2	NA
		*Achromobacter xylosidans*/MRSA^*c*^	1	NA
OHSU bronchiectasis (10)	Bronchiectasis (chronic)	Pseudomonas aeruginosa	10	NA
URMC S. aureus (15)	Osteomyelitis and septic arthritis (chronic)	Staphylococcus aureus	15	NA

a DHMC, Dartmouth-Hitchcock Medical Center; OHSU, Oregon Health & Science University; URMC, University of Rochester Medical Center. NA, not available.

b Others, from the DHMC bacteremia cohort, include coagulase-negative *Staphylococcus* (9), Klebsiella oxytoca (5), Enterobacter cloacae (3), Proteus mirabilis (3), Corynebacterium striatum (2), Pasteurella multocida (2), Streptococcus pneumoniae (2), Bacteroides fragilis (1), beta-hemolytic streptococci (1), Candida tropicalis (1), Clostridioides difficile (1), Corynebacterium glucuronolyticum (1), Enterococcus avium (1), *Fusobacterium* species (1), Klebsiella aerogenes (1), Listeria monocytogenes (1), *Micrococcus* species (1), Morganella morganii (1), *Prevotella* species (1), Streptococcus mitis (1), and Streptococcus sanguinis (1).

c MRSA, methicillin-resistant Staphylococcus aureus.

**FIG 2:**
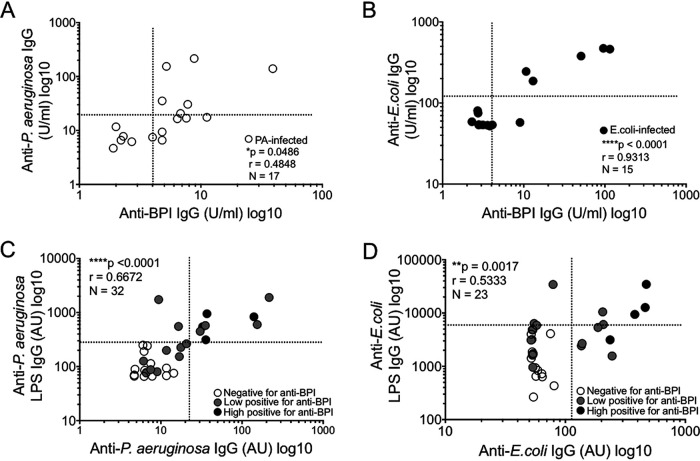
Anti-BPI IgG correlates with anti-P. aeruginosa and anti-E. coli IgG responses in bacteremia patients. (A and B) Serum anti-BPI IgG titers were correlated with levels of serum anti-P. aeruginosa IgG (*r* = 0.4848; *, *P* = 0.0486) (A) and serum anti-E. coli IgG (*r* = 0.9313; ****, *P* < 0.0001) (B). (C) Serum anti-P. aeruginosa protein extracts were correlated with anti-P. aeruginosa LPS IgG levels (*n* = 16, *r* = 0.6672; ****, *P* < 0.0001). (D) Serum anti-E. coli protein extracts were correlated with anti-E. coli LPS IgG (*n* = 16, *r* = 0.5333; **, *P* = 0.0017). (C and D) Empty circles represent samples negative for anti-BPI IgG by ELISA, gray circles represent low positives (≤39 AU, below 7th octile), and black circles represent high positives (>39 AU, top octile [8th]) (*n* = 16). Associations were determined by Pearson correlation analysis of the Duke bacteremia cohort (*n* = 32). The positive cutoffs, determined as the mean values for healthy controls plus 2SD (*n* = 53), represented by dashed lines. were as follows: anti-BPI, >4 AU; anti-P. aeruginosa IgG, >22 U/ml; anti-E. coli IgG, >112 U/ml; anti-P. aeruginosa LPS IgG, >283.6 U/ml; anti-E. coli LPS IgG, >5,933.8 U/ml.

The presence of anti-BPI autoantibodies in the patient sera was strongly associated with increased levels of antibodies targeting P. aeruginosa and E. coli (Fig. 1B and C). Consistent with this, we saw a correlation between anti-BPI autoantibody and antibacterial antibody titers (Fig. 2A and B). Interestingly, this correlation was comparable to, or exceeded that, seen with IgG responses to GNB protein extracts and their respective LPS (Fig. 2C and D).

### Consecutive cohort analysis of GNB and GPB bacteremic patients.

The strong relationship between BPI antibody autoreactivity and bacteremia was observed in a retrospective cohort restricted to GNB (Fig. 1 and 2). Thus, it is unclear if the development of autoantibodies to BPI in bacteremia is restricted to P. aeruginosa and E. coli, i.e., to GNB. Moreover, the retrospective nature of the sample collection made it impossible to link the time of serum sampling relative to the onset of bacteremia. To address these issues, a prospective collection of sera from patients with confirmed bacteremia (*n* = 154; see Dartmouth-Hitchcock Medical Center [DHMC] cohort characteristics in Table 1) was undertaken at DHMC. Anti-BPI IgG antibodies were detected in 14.9% (23/154) of serum samples from bacteremic patients (Table 2). Anti-BPI reactivity was seen in 17.2% (5/29) and 13.3% (8/60) of sera from patients with E. coli and S. aureus, respectively. P. aeruginosa was an infrequent cause of bacteremia in this cohort; hence, sera from patients with P. aeruginosa were uncommon (12/154), with 33.3% (4/12) exhibiting anti-BPI antibodies. Slightly lower mean titers of IgG anti-BPI antibodies were seen in GNB bacteremia than in bacteremia from Gram-positive bacteria (GPB) (mean number of AU, 87 versus 127, respectively). Mean anti-BPI autoreactivities were comparable (157 ± 185 AU versus 134 ± 59.3 AU) in S. aureus- and P. aeruginosa-infected patients, respectively, while lower (20 to 89 AU) titers were seen in sera from patients with E. coli and other bacteria (Table 2). No relationship was seen between anti-BPI IgG responses and IgG antibody titers specific for E. coli or P. aeruginosa in patient subsets (*n* = 87 and *n* = 152, respectively) from this cohort, although a trend (*P* = 0.0514) was seen with P. aeruginosa (see Fig. S2 in the supplemental material).

**TABLE 2:** Anti-BPI IgG positivity in the DHMC bacteremia cohort, separated into Gram-negative and Gram-positive groups^*a*^

Sample group and/or organism	No. of anti-BPI-positive samples	Total no. of samples	% Positive samples	Avg anti-BPI of positive samples (AU) (SD)	% Positive	Avg anti-BPI of positive samples (AU) (SD)
All samples	23	154	14.9	106 (123)	14.9	106 (123)
Gram-negative samples						
Escherichia coli	5	29	17.2	84.7 (78.9)		
Klebsiella pneumoniae	2	10	20.0	27.2 (5.27)		
Pseudomonas aeruginosa	4	12	33.3	134 (59.3)	17.9	87.0 (70.8)
Proteus mirabilis	1	3	33.3	28.4 (0)		
Others	0	13	0.00	NA		
						
Gram-positive samples						
Staphylococcus aureus	8	60	13.3	157 (185)		
Enterococcus faecalis	1	3	25.0	19.6 (0)		
Streptococcus pneumoniae	1	2	50.0	34.4 (0)	12.8	127 (164)
Corynebacterium glucuronolyticum	1	1	100	88.5 (0)		
Others	0	20	0.00	NA		
						
Candida tropicalis (yeast)	0	1	0.00	NA	0.00	0 (0)

a For definitions of “Others” in column 1 and NA, see footnotes *a* and *b* in Table 1.

### Autoreactivity to BPI in acute versus chronic S. aureus infection.

**FIG 3:**
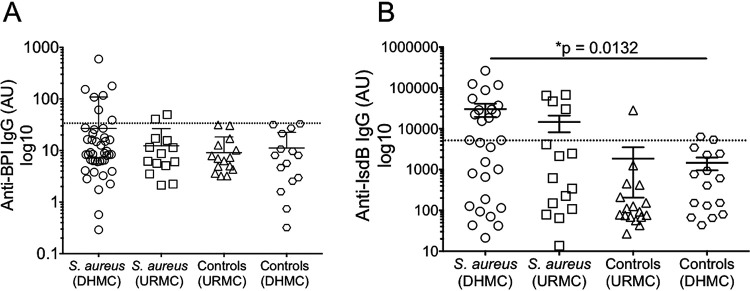
BPI autoreactivity occurs in both acute and chronic S. aureus infections. (A) Anti-BPI IgG titers were detected by ELISA in the DHMC S. aureus bacteremia (*n* = 60) and URMC chronic S. aureus (bacteremic arthritis/osteomyelitis) infection (*n* = 15) cohorts. Two control groups represent healthy controls from URMC (*n* = 17) and DHMC (*n* = 15). Anti-BPI IgG-positive samples are indicated by values of >33.81 AU; the positive cutoff was determined as the mean result for healthy controls (HC) plus 2SD and is represented by a dashed line. (B) Anti-IsdB IgG titers were detected by ELISA in a subpopulation of DHMC S. aureus bacteremia (*n* = 19) and URMC chronic S. aureus infection (*n* = 15) cohorts. Two control groups represent healthy controls from URMC (*n* = 17) and DHMC (*n* = 16). Anti-IsdB IgG-positive samples are indicated by values of >5,508.83 AU; the positive cutoff was determined as the mean value for healthy controls (HC) plus 2SD (*n* = 16) and is represented by a dashed line.

The induction of anti-BPI IgG in 13.3% (8/60) of patients with S. aureus bacteremia prompted further examination as to whether more chronic infection with this pathogen might lead to further induction of autoreactivity to BPI. We compared BPI autoreactivity in patients with S. aureus bacteremia with that in patients with S. aureus arthritis and osteomyelitis (23). Anti-BPI autoantibodies were found in 2/15 (13.3%) patients with chronic S. aureus infection (S. aureus [URMC]) (Fig. 3A), approximating the rate seen in acute S. aureus bacteremia (S. aureus [DHMC]) (Fig. 3A; Table 2). These two cohorts had comparable levels of anti-IsdB IgG titers, a marker of S. aureus infection (23) (Fig. 3B). The presence of anti-IsdB IgG did not correlate with anti-BPI IgG (data not shown), suggesting that autoreactivity to BPI is not associated with a humoral immune response to S. aureus, in contrast to our previous observations in chronic P. aeruginosa infection in CF and BE patients (8–10).

### Temporal induction and avidity of anti-BPI IgG responses.

**FIG 4:**
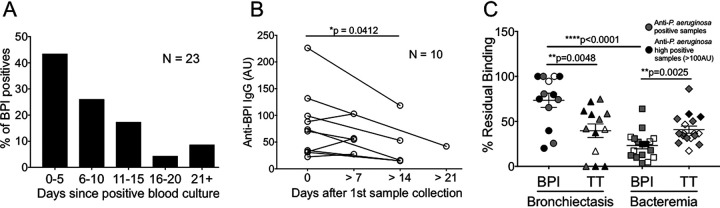
Anti-BPI IgG antibodies in bacteremic patients arise rapidly and are of low avidity. (A) The percentage of anti-BPI IgG-positive patients who became positive 0 to 5, 6 to 10, 11 to 15, 16 to 20, and >21 days after a positive blood culture for infected organisms was studied in the DHMC bacteremia cohort that was positive for anti-BPI IgG (*n* = 23). Each of the patients was sampled at the indicated time interval. (B) A subset (*n* = 10) of DHMC bacteremia patient sera was tracked over time (>21 days), and anti-BPI IgG titers were evaluated by ELISA. Circles connected by lines represent samples from the same patient. Statistical significance was determined by a paired Student's *t* test relative to the day 0 sample group (*, *P* < 0.05). (C) The avidity of anti-BPI antibodies from two patient cohorts (*n* = 13 for bronchiectasis, *n* = 17 for DHMC bacteremia) was compared to antibody avidity to recall antigen tetanus toxoid (TT; 57.05% ± 4.607% and 40.79% ± 3.939% [mean ± SEM], respectively, for the BE and DHMC bacteremia cohorts), using a 3 M NaSCN elution, by a paired Student's *t* test (**, *P* < 0.01). Gray symbols represent anti-P. aeruginosa positive samples; black symbols represent anti-P. aeruginosa high positive samples (>100 AU). Three bronchiectasis samples show no residual binding to TT. Statistical difference in avidity of anti-BPI IgG in BE and bacteremia cohorts was determined by Student's *t* test (****, *P* < 0.0001).

One notable feature of anti-BPI IgG induction in bacteremic patients was its appearance at the time of the first serum collection, as demonstrated by examination of the kinetics of the presence of these antibodies in the first serum sample relative to the time of positive blood culture (Fig. 4A). In the DHMC bacteremia cohort, 43.5% of anti-BPI IgG-positive patients (*n* = 23) were positive within the first 5 days of testing positive for infection (Fig. 4A), suggesting that anti-BPI antibody was induced early during the course of infection. Tracking a subset of the DHMC bacteremia cohort over time revealed that anti-BPI autoantibody titers significantly decreased with time (Fig. 4B).

We report higher BPI protein levels in bacteremia patients than in healthy controls (Fig. S3A). BPI protein and anti-BPI IgG positivity detected in sera did not correlate with age and was not associated with sex in the DHMC bacteremia patient cohort (Fig. S3B to D). Furthermore, there was no correlation between serum BPI or myeloperoxidase (MPO) protein levels (markers for neutrophil activation) and anti-BPI IgG positivity (Fig. S4), suggesting that anti-BPI IgG was not related to the magnitude of neutrophil activation (24, 25).

Notably, we have previously reported that IgG autoreactivity to BPI found in CF cohorts is of high avidity, equivalent to that seen for P. aeruginosa antigens and tetanus toxoid (TT), a control recall antigen (10). Evaluating the avidity of anti-BPI IgG binding with sodium isothiocyanate elution (10, 26, 27) revealed high avidity binding in sera from patients with BE, as previously seen with CF sera (10), with avidity equivalent to that of anti-TT IgG (Fig. 4C). In contrast, IgG responses to BPI in patients with bacteremia all exhibited significantly lower avidity than that of anti-TT IgG (*P* = 0.0025) (Fig. 4C). Bronchiectasis patients exhibited markedly higher anti-BPI avidity than patients with bacteremia (Fig. 4C). When antibodies to P. aeruginosa are used as an index of humoral immunity (chronicity) to P. aeruginosa infection, no relation to avidity of antibodies to BPI was seen in bronchiectasis or bacteremic patients; i.e., high antibody titers to P. aeruginosa are detected equally in patients with low- or high-avidity antibodies to BPI (Fig. 4C).

## DISCUSSION

Chronic airway infection with P. aeruginosa is associated with the development of autoantibodies to BPI in bronchiectasis, cystic fibrosis, and chronic obstructive pulmonary disease (1–4, 8, 9, 17, 28–30). Previous studies have reported the ability of anti-BPI antibody responses to compromise function. Affinity-purified anti-BPI autoantibodies inhibited bacterial phagocytosis *in vitro* (31), suggesting a role of these autoantibodies against opsonization. The C‐terminal region of the BPI molecule was reported to be crucial for opsonization and disposal of Gram‐negative bacteria and lipopolysaccharides (32, 33). BPI-mediated killing of E. coli was impaired by anti-BPI antibodies from pediatric cystic fibrosis patients (34), and neutrophil-mediated killing of E. coli was inhibited by the addition of neutralizing anti-BPI serum from rabbit (35). In inflammatory bowel disease and Wegener's granulomatosis patients, anti-BPI antibodies were shown to impair the antibiotic activity of BPI protein (36, 37). BPI antibody autoreactivity is associated with increased morbidity and mortality in these diseases (9, 10, 17, 28–30). This presumably occurs through autoantibody-dependent compromise of innate immune responses. One model for the association of P. aeruginosa with anti-BPI IgG is molecular mimicry, where P. aeruginosa has a particular ability to induce antibodies directed against BPI. To investigate this model we asked the following questions. (i) Can acute P. aeruginosa infection lead to anti-BPI IgG induction, or is chronic infection required? (ii) Does autoreactivity to BPI arise during acute infection with other GNB (e.g., E. coli) or even GPB (e.g., S. aureus)? (iii) Are anti-BPI IgG responses in various infections qualitatively different and mediated by pathophysiologic pathways?

We addressed the first question by examining the frequency of anti-BPI IgG antibodies in a cohort of patients with P. aeruginosa bacteremia (Duke cohort). The frequency of BPI antibody autoreactivity in this cohort was nearly 65%, compared to ∼43% previously reported in CF cohorts and ∼38 to 45% previously reported in BE cohorts harboring chronic P. aeruginosa infections (9, 10). Surprisingly, IgG antibody autoreactivity to BPI was also seen in about 47% of sera from patients with E. coli bacteremia. Thus, the development of autoantibodies to BPI occurred in Gram-negative bacteremia with high incidence but was not restricted to P. aeruginosa infection.

We examined this result in a prospective confirmatory cohort of bacteremic patients. BPI IgG antibody autoreactivity was evident in 14.9% and was present in both GNB and GPB infections. There were only 12 patients with P. aeruginosa in the cohort, 4 of whom (33.3%) had anti-BPI IgG. Since the total numbers of P. aeruginosa-positive patients were few, we conclude that GNB and GPB, notably E. coli, P. aeruginosa, and S. aureus, can induce anti-BPI autoreactivity. Anti-BPI IgG rates in acute and chronic S. aureus infections were comparable, both at 13.3%, further suggesting that BPI autoantibody induction is not restricted to chronic bacterial infections. Additionally, we found that anti-BPI IgG in bacteremia, bronchiectasis, and cystic fibrosis patients were all of the IgG1, not the IgG2, subclass (Fig. S5). These data suggested that antibody responses arise from soluble protein antigens and membrane proteins rather than due to cross-reactivity with bacterial capsular polysaccharide antigens (38).

Thus, both the Duke bacteremia and DHMC bacteremia cohorts demonstrated that BPI autoantibody generation can associate with acute infections from bacteria other than P. aeruginosa. It is unclear why the autoantibody frequencies were so different in selected versus prospective cohorts. In this regard, key information as to the level of acute illness or source of bacteremia in the retrospective Duke cohort might be useful. What is clear is that a prospective analysis of bacteremic patients exhibited IgG anti-BPI responses within days of the onset of bacteremia. In addition, there was no evidence of an increasing antibody titer over time, as one would expect with a memory B cell response. These data suggested that the development of autoantibodies to BPI occurring with bacteremia was not due to follicle-dependent B cell maturation, where IgG responses occur in a T cell-dependent manner in germinal centers 7 to 21 days following initial antigen exposure. The rapid induction of anti-BPI IgG with bacteremia suggests that T cell-dependent follicular maturation of IgG-bearing B cells characterized by affinity maturation was unlikely. This notion was supported by the demonstration that anti-BPI antibody responses in bacteremia were of low avidity, in contrast to those seen in CF and BE (10) (Fig. 4C).

These data indicate that BPI antibody autoreactivity is qualitatively different as a function of disease (bacteremia versus P. aeruginosa airway infection without bacteremia), and it is unclear which type predominates in other disease states such as inflammatory bowel disease, where BPI autoantibodies are also seen (36, 39). Thus, there are likely parallel paths to the induction of anti-BPI antibody autoreactivity, one that involves low-avidity responses such as those seen with marginal zone B cells (40). Our findings suggest that this pathway predominates in acute bacteremia. There has been a report of BPI binding to both LPS and lipopeptides on S. aureus, enhancing bacterial lipoprotein recognition (41). The bacteremia presumably facilitates delivery of BPI complexed with GNB/GPB to the marginal zone of the spleen, resulting in activation of their polyreactive B-cell receptor (BCR) and Toll-like receptor (TLR) to result in low-avidity IgG autoantibodies. Unresolved is the mechanism of the very strong association of chronic P. aeruginosa with high-avidity IgG responses to BPI. Interestingly, we show that autoantibodies to neutrophil antigens other than BPI, such as PR3, may arise in bacteremia (see Fig. S6A in the supplemental material). However, anti-PR3 IgG levels do not track together with IgG reactivity to BPI, P. aeruginosa, or E. coli, suggesting differential specificities and/or mechanisms leading to autoreactivity (Fig. S6B to D). While these findings indicate that BPI antibodies are developed at a high frequency in patients with chronic P. aeruginosa infection of the airway, it does not distinguish which component (the airway, the organism, or its chronicity) leads to this outcome. Similarly, one cannot distinguish whether initial marginal zone B cell responses played a role in developing high-avidity IgG responses to BPI. Finally, we are unable to determine if these two pathways of antibody autoreactivity differentially wane over time.

In summary, these data demonstrate that acute P. aeruginosa infection can lead to development of autoantibodies to BPI, without a requirement for a chronic infection state. During acute infection, BPI autoreactivity arises not only in the presence of other GNB (e.g., E. coli) but also in the presence of GPB (e.g., S. aureus). The evidence of decreased BPI autoreactivity titers over time, together with the low anti-BPI IgG avidity shown in the bacteremia cohort, suggests that BPI autoreactivity arises in a T cell-independent manner without follicle-dependent B cell maturation. Understanding how BPI autoantibodies develop is an essential step in identifying the pathogenic role of these autoantibodies in both chronic and acute infections.

## MATERIALS AND METHODS

### Patient cohorts.

Serum samples were obtained from six different deidentified adult (≥18-year-old) patient cohorts: (i) Duke bacteremia (*n* = 32), (ii) Dartmouth-Hitchcock Medical Center (DHMC) bacteremia (*n* = 154), (iii) DHMC bronchiectasis (*n* = 3), (iv) Oregon Health & Science University (OHSU) bronchiectasis (*n* = 10), (v) University of Rochester Medical Center (URMC) chronic S. aureus arthritis and osteomyelitis patients (*n* = 15) and healthy controls (*n* = 17), and (vi) DHMC healthy controls (*n* = 53). DHMC healthy controls were employees who donated serum. The information on patient cohorts is summarized in Table 1. Specimens from the Duke bacteremia cohort consisted of residual serum specimens (typically within 3 days of bacteremia) from inpatients with E. coli or P. aeruginosa bacteremia. Specimens from the DHMC bacteremia cohort consisted of residual serum specimens (serum collection was typically within 5 days of bacteremia) and excluded some coagulase-negative *Staphylococcus*-positive blood cultures to minimize blood culture contaminants. The Duke Institutional Review Board (IRB) and the Dartmouth Hitchcock Health Institutional Review Board (DHH-IRB) approved the use of residual patient specimens for this study. Written informed consent was obtained from patients or their legally authorized representatives. In the event that a patient died before enrollment into the Duke Bloodstream Infection Biorepository, clinical samples were ascertained using Decedent Research approval. Patient samples were deidentified.

### Antibody and protein detection by ELISA.

Two assays were developed to measure serum anti-BPI IgG detection by ELISA and were deemed comparable. For Duke bacteremia patient serum samples, antibody reactivity to BPI was determined by a commercially available anti-human BPI ELISA kit, according to the manufacturer’s instructions (Orgentec Diagnostika GmbH). The positive reactivity threshold was established as the mean reactivity of healthy controls (*n* = 16) plus 2 standard deviations (SD) (>6 U/ml). A standard curve was established using serial dilutions from a known positive serum sample, and arbitrary units were calculated. A negative control using a known negative serum sample was included. Strong correlation (*r* = 0.9001, *P* < 0.0001) was shown between Orgentec’s ELISA kit and an in-house BPI-coated ELISA on a subset of DHMC bacteremia samples (see Fig. S1 in the supplemental material), and we previously showed that these assays give comparable results (8). Hence, for other patient cohorts, antibody reactivity to BPI was determined by coating an ELISA plate with 10 μg/ml of BPI (Athens Research and Technology) for 2 h at 37°C. The plates were then washed (phosphate-buffered saline [PBS] plus 0.05% Tween 20) and blocked overnight at 4°C in PBS plus 1% bovine serum albumin (BSA). Patient sera (1:100 dilution in PBS plus 1% BSA) were added and incubated for 2 h at room temperature (RT). Goat anti-human peroxidase-labeled IgG F(ab′)2 (1:50,000 in PBS plus 1% BSA; Bio-Rad, USA) was added and incubated for 1 h at RT. Reactivity was detected using chromogenic substrate (R&D Systems). Absorbance was determined at 450 nm using an ELISA reader (Epoch; BioTek).

To measure anti-P. aeruginosa, anti-E. coli, and anti-S. aureus antibody titers, ELISA plates (R&D Systems) were coated with 10 μg/ml P. aeruginosa (PA14 wild type), E. coli, or S. aureus lysate in PBS for 2 h at 37°C. Lysates were generated by repeated (three) freeze-thaw cycles of overnight bacterial cultures (P. aeruginosa strain PA14, E. coli strain GN02546, protein A-deficient S. aureus strain). The plates were then washed and blocked as mentioned above. Patient sera (1:1,000 dilution in PBS plus 1% normal goat serum [NGS]) were added and incubated for 1 h at RT. The ELISA was developed as described above. IgG1 (Invitrogen) and IgG2 (SouthernBiotech) reactivities to BPI were determined using a similar in-house ELISA method. Anti-LPS IgG antibodies were detected by ELISA as described above, except that plates were coated with 1 μg/ml P. aeruginosa or E. coli LPS (Sigma-Aldrich), and patient sera were diluted 1:100. Reactivity to PR3 was determined using a commercial kit (Inova Diagnostics) in accordance with the manufacturer’s instructions. BPI and MPO protein in patient serum were measured using a commercial sandwich ELISA DuoSet kit (R&D Systems) by following the manufacturer’s instruction.

### Serum anti-iron-regulated surface determinant protein B (IsdB) antibody (IgG) detection by ELISA.

To measure anti-IsdB antibody titers, ELISA plates (R&D Systems) were coated with 5 μg/ml avidin in PBS overnight at 4°C. The plates were then washed and blocked in PBS plus 1% BSA for 2 h at RT. Biotinylated IsdB protein (generously provided by Edward Schwarz, URMC) was incubated (5 μg/ml) in the plate for 2 h at RT. The plates were then washed, and patient serum was added and incubated for 1 h at RT. The ELISA was developed as described above.

### Antibody avidity assays.

IgG avidity of antibodies specific for BPI (Athens Research) and tetanus toxoid (TT; Tenivac) was examined by ELISA elution assays as previously described (26, 27). Serial serum dilutions that resulted in 50% maximum binding to antigens (1:500 for BPI; 1:250 for TT) were identified. Dose-dependent effects of chaotropic agent thiocyanate (NaSCN; 0 to 5 M) on elution of anti-BPI IgG were examined to determine the NaSCN concentration necessary to elute 50% of the antibody. Antibody avidity was calculated as the percentage of the amount of residual antibodies bound to the coated antigen after NaSCN elution, relative to the amount of antibodies prior to elution, and is presented as the percentage of residual binding (10). The avidity of anti-BPI IgG from 13 BE (10 OHSU samples and 3 DHMC samples) and 17 bacteremia (DHMC) serum samples was compared to antibody avidity to recall antigen TT, using 3 M NaSCN eluent, based on titration studies which demonstrated that this concentration reduced anti-BPI antibody binding by ∼50% (10). Anti-BPI autoantibody positivity was used as the criterion in choosing a subset of samples to run in an avidity assay, both for BE and bacteremia cohorts.

### Statistical analysis.

Data were analyzed using GraphPad Prism 7 software (GraphPad Software Inc.). Significant differences between sample populations were determined by Student's *t* test with Welch’s correction (two groups). Pearson coefficient correlation analysis was used to determine the strength of the relationship between antibody titers. *P* values of <0.05 were considered significant.

## Supplementary Material

Supplemental file 1
