# Supplementary material for: Low-Avidity Autoantibodies against Bactericidal/Permeability-Increasing Protein Occur in Gram-Negative and Gram-Positive Bacteremia
Source: Infect Immun. 2020 Sep 18;88(10):e00444-20. doi: 10.1128/IAI.00444-20 (PMC7504969; doi:10.1128/IAI.00444-20)
Supplement: Supplemental file 1 [file IAI.00444-20-s0001.pdf]

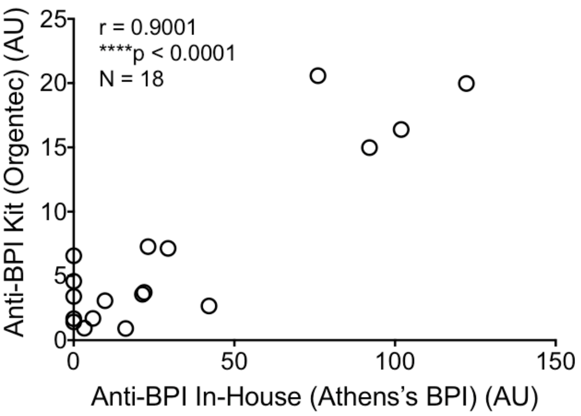


**Supplemental Figure 1: Correlation between anti-BPI measurements by commercial and in-house ELISA in DHMC bacteremia cohort.** Correlation between two anti-BPI ELISAs, one coated with Athens Research and Technology's BPI, compared to Orgentec's anti-BPI ELISA kit; r=0.9001; ****p<0.0001; n=18 from a subset of DHMC bacteremia cohort. Associations were determined by Pearson correlation analysis.

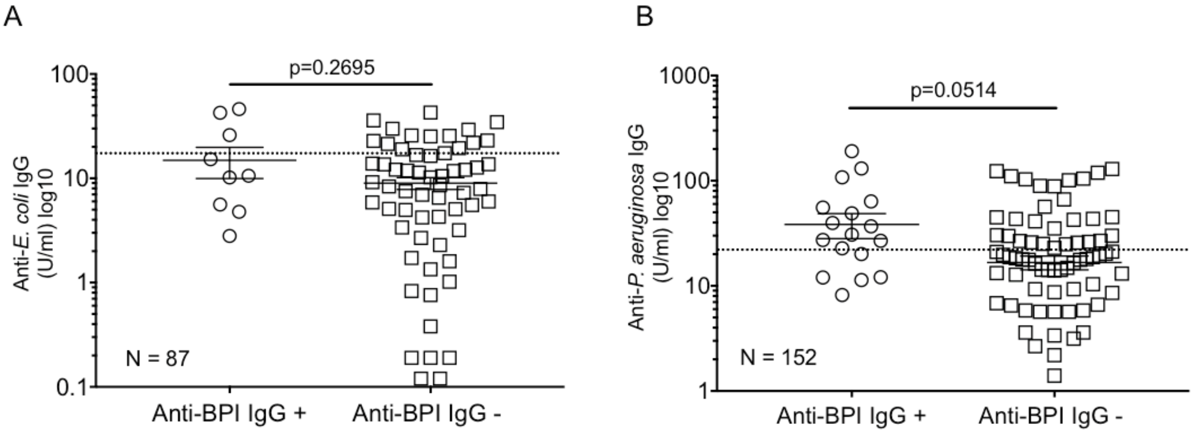


**Supplemental Figure 2: BPI reactivity does not correlate with either *E. coli* or *P. aeruginosa* IgG reactivity in DHMC bacteremia cohort.** Anti-BPI IgG positivity, determined by ELISA, does not show association with antibody reactivity to **(A)** *E. coli* (GN02546 lysate) or **(B)** *P. aeruginosa* (PA14 lysate) in DHMC bacteremia cohort; reactivity to *E. coli* and *P. aeruginosa* (n=87 and n=152 respectively) determined by ELISA (positive cutoff of >17.12 U/ml and >22 U/ml respectively, represented by dashed line). Statistical significance was determined by Student's t-test, *p < 0.05.

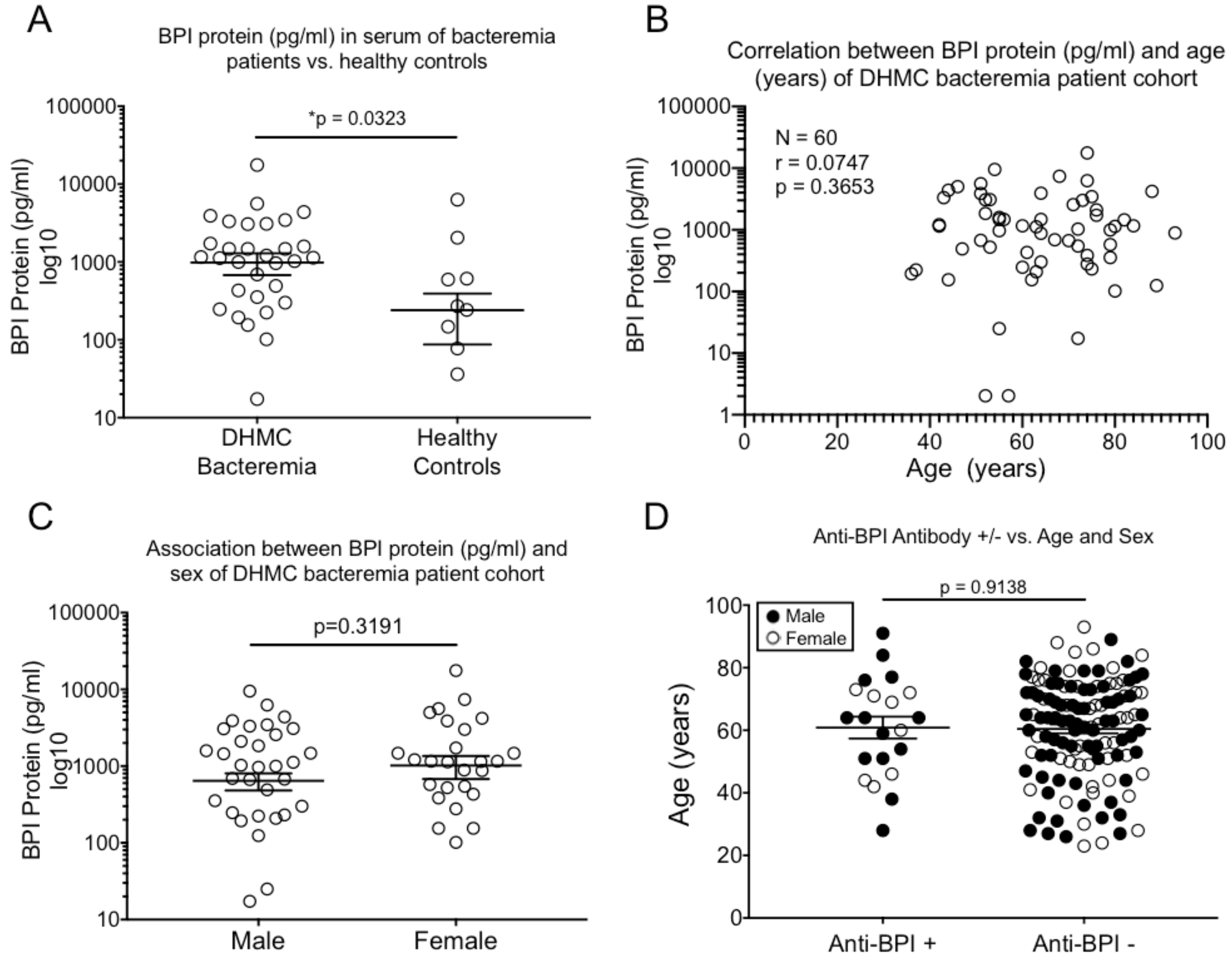


**Supplemental Figure 3: BPI protein and anti-BPI IgG positivity do not correlate with age or associate with sex in the DHMC bacteremia patient cohort. (A)** BPI protein titers were detected by sandwich ELISA in DHMC bacteremia (n=31) and healthy control (n=9) cohorts. Statistical significance was determined by Student's t-test, *p < 0.05. **(B)** Correlation between BPI protein and age of bacteremia patients; r=0.0747; p=0.3653; n=60 from a subset of DHMC bacteremia cohort. Associations were determined by Pearson correlation analysis. **(C)** BPI protein titers were analyzed against sex (n=32 for male, n=26 for female) of DHMC bacteremia patient cohort. Statistical significance was determined by Student's t-test, *p < 0.05. **(D)** Age of DHMC bacteremia patient cohort was analyzed against anti-BPI IgG positivity (n=149). Filled symbols represent male patient samples; unfilled symbols represent female patient samples. Statistical significance was determined by Student's t-test, *p < 0.05.

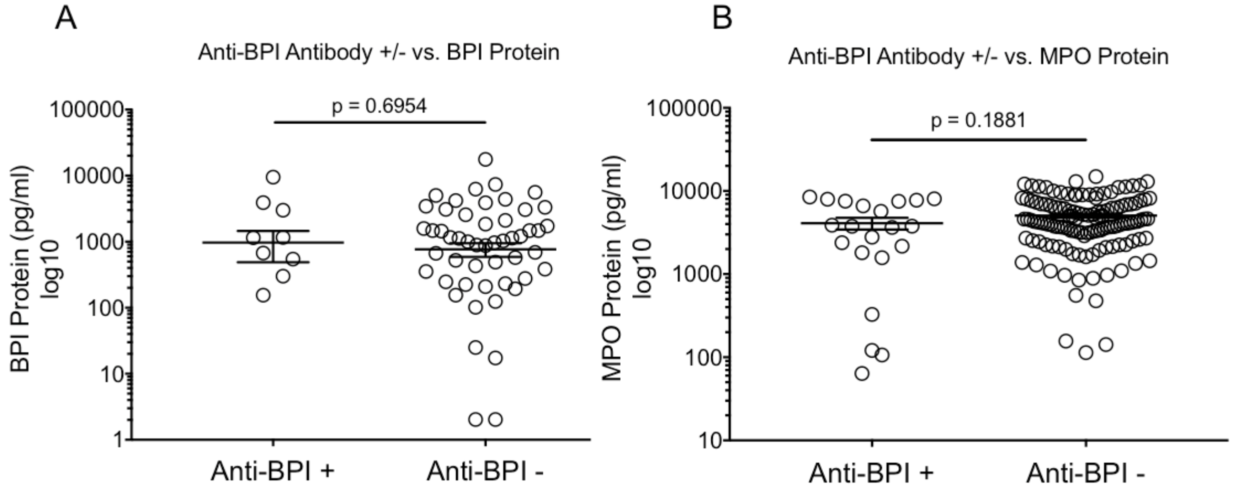


**Supplemental Figure 4: No association is observed between BPI or MPO protein and anti-BPI IgG positivity. (A)** BPI (n=60) and **(B)** MPO (n=149) protein titers were detected by sandwich ELISA in DHMC bacteremia cohort, and analyzed against anti-BPI IgG positivity. Statistical significance was determined by Student's t-test, $^*p < 0.05$.

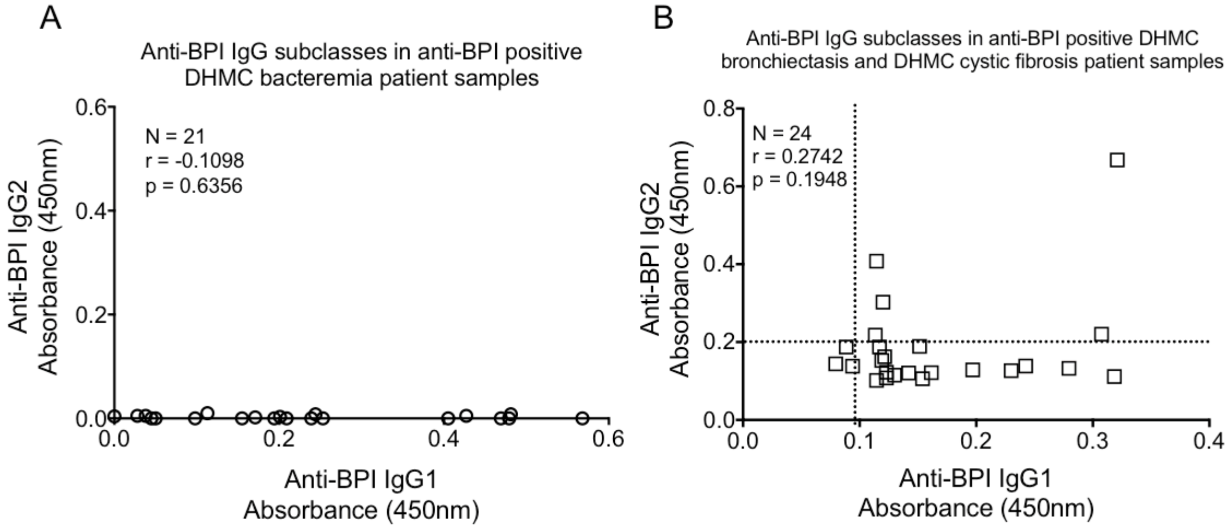


**Supplemental Figure 5: Anti-BPI IgG in bacteremia, bronchiectasis, and cystic fibrosis patients are of IgG1 subclass. (A)** Anti-BPI IgG subclass reactivity, IgG1 and IgG2, were analyzed in DHMC bacteremia patient samples (n=21). **(B)** Anti-BPI IgG subclass reactivity, IgG1 and IgG2, were analyzed in DHMC bronchiectasis and cystic fibrosis patient serum samples (n=24). Positive cutoffs were determined as mean of healthy controls +2SD (n=20), represented by dashed lines. Associations were determined by Pearson correlation analysis.

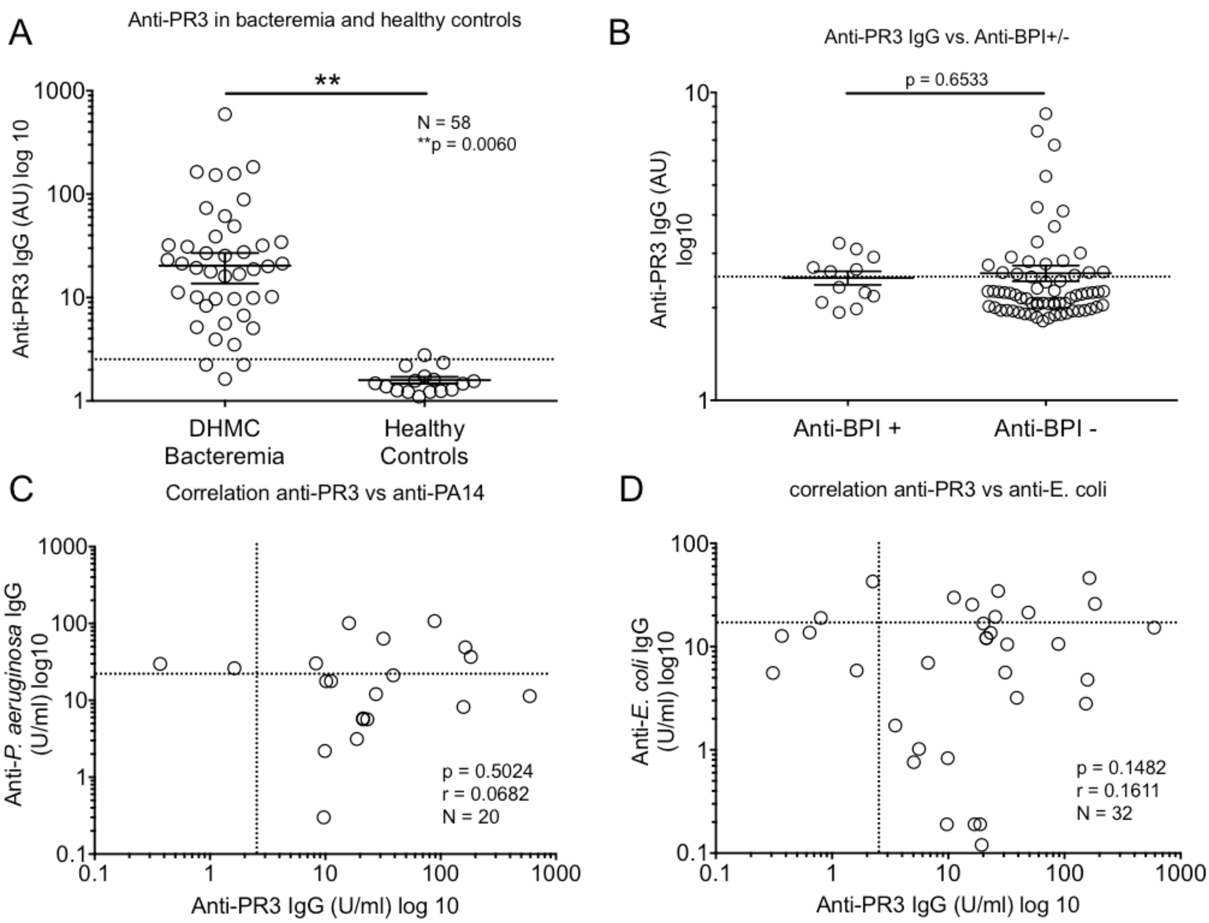


**Supplemental Figure 6: Autoantibodies to other neutrophil antigens may arise, but do not track together with anti-BPI IgG positivity, *P. aeruginosa*, or *E. coli*. (A)** Anti-PR3 IgG titers were detected by ELISA in DHMC bacteremia (n=42) and healthy control (n=16) cohorts. Statistical significance was determined by Student's t-test, **p < 0.01. **(B)** Anti-PR3 IgG titers were analyzed against anti-BPI IgG positivity in bacteremia patients (n= 80). Statistical significance was determined by Student's t-test, *p < 0.05. No correlation was seen between anti-PR3 IgG titers and **(C)** anti-*P. aeruginosa,* or **(D)** anti-*E. coli* IgG of bacteremia patients. Associations were determined by Pearson correlation analysis.
